# Controlled *In Meso* Phase Crystallization – A Method for the Structural Investigation of Membrane Proteins

**DOI:** 10.1371/journal.pone.0035458

**Published:** 2012-04-19

**Authors:** Jan Kubicek, Ramona Schlesinger, Christian Baeken, Georg Büldt, Frank Schäfer, Jörg Labahn

**Affiliations:** 1 QIAGEN GmbH, Hilden, Germany; 2 Molecular Biophysics, Institute of Structural Biology and Biophysics (ISB-2), Research Center Jülich, Jülich, Germany; University of Oulu, Finland

## Abstract

We investigated *in meso* crystallization of membrane proteins to develop a fast screening technology which combines features of the well established classical vapor diffusion experiment with the batch meso phase crystallization, but without premixing of protein and monoolein. It inherits the advantages of both methods, namely (i) the stabilization of membrane proteins in the meso phase, (ii) the control of hydration level and additive concentration by vapor diffusion. The new technology (iii) significantly simplifies *in meso* crystallization experiments and allows the use of standard liquid handling robots suitable for 96 well formats. CIMP crystallization furthermore allows (iv) direct monitoring of phase transformation and crystallization events. Bacteriorhodopsin (BR) crystals of high quality and diffraction up to 1.3 Å resolution have been obtained in this approach. CIMP and the developed consumables and protocols have been successfully applied to obtain crystals of sensory rhodopsin II (SRII) from *Halobacterium salinarum* for the first time.

## Introduction

Although one third of a cell's proteome represents membrane proteins, they constitute a distinct minority with regard to known 3-dimensional structures at atomic resolution. Methods developed for soluble protein crystallization might be often inefficient for membrane proteins. This situation is especially bothersome as the natural entry points to a cell are membrane proteins and their assemblies: The lack of knowledge of membrane protein structures translates directly into lack of knowledge of this single most important group of biomedical targets and their mechanisms of activity.

The small number of membrane protein structures known can be directly traced back to problems in obtaining membrane protein crystals for structural investigations. Membrane proteins are difficult to crystallize using the methods that had been developed and very successfully applied for soluble proteins. For these proteins, the employment of the currently widely used automated dispensing systems to set up vapor diffusion crystallization experiments [1], and the availability of thousands of pre-made crystallization solutions boosted the number of successful crystallization projects. Obviously, the multitude of different conditions does not simply reflect the requirement to decrease the solubility of the protein to induce crystallization, but the necessity to stabilize certain states of the target protein by interaction with components of these screens [2]. For detergent-solubilized membrane proteins, similar progress has not been made. Clearly, better methods for crystallization or better screens or both are required to increase the chance to obtain well diffracting membrane protein crystals.

The development of the lipidic cubic phase (LCP) crystallization [3], [4] introduced a new approach using monoolein-water based mesophases [5] that accommodate membrane proteins better than the conventional water-detergent systems: designed as a batch method, the classical *in cubo* crystallization experiment is cumbersome to perform [3]. It requires extensive manual labor like weighing of mg quantities of monoolein and salt for every single crystallization experiment. This is detrimental to high throughput screening of available conditions and excludes the application of automated liquid handling systems. Attempts to increase the throughput of cubic phase batch crystallization procedures by the use of special equipment that allows dispensing of a premixed monoolein-protein-water paste [6], [7] have been used but high costs for the required extra dispensing system are involved.

**Figure 1:**
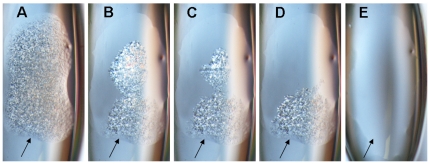
Monoolein/water self-organization into mesophase: time course of mesophase formation. 900 nl water was added to a protein well of a crystallization micro plate coated with 132 µg monoolein (MO). The optical properties of the forming phases were examined under a polarization microscope over time. **A**, t = 1 min, **B**, t = 5 min **C**, t = 21 min, **D**, t = 22 min, **E**, t = 39 min Note: Arrows indicate the lower boundary of the forming isotropic meso-phase.

In contrast to the method that emphasizes active mixing [8] and subsequent dispensing of a paste of meso phase either manually by a syringe or with special dispensing systems, the pioneer experiments [3] imply that no active mixing of aqueous protein solution and dry monoolein is required to obtain the cubic phase with embedded membrane protein. Another major difference between these approaches is the way by which crystallization is induced: when using protein-monoolein paste [8] this is achieved by adding a precipitating (additive) solution, whereas the approach in the Landau method was the addition of solid salt in order to lower the free water content. In our method, the protein incorporation into the lipidic phase occurs passively (Figure 1), whereas the crystallization is induced by increase of the protein and the additive (precipitant) concentration through dehydration by vapor diffusion.

**Figure 2:**
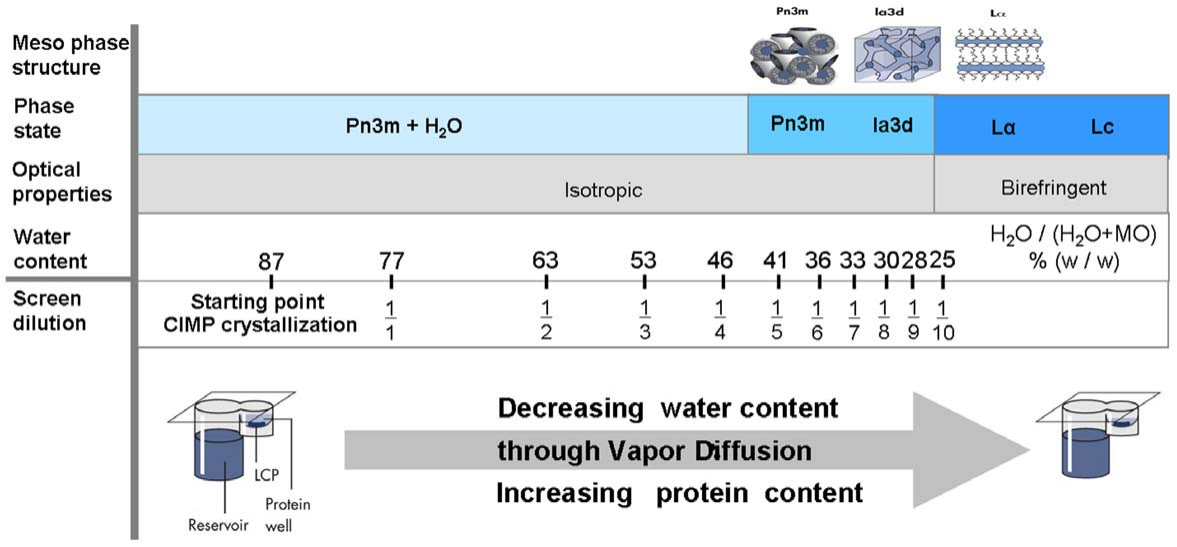
The monoolein/water isotherm at 22°C. With increasing water content, the layered lamellar phases (Lc, Lα), cubic phases (Ia3d, Pn3m) and the phase Pn3m + water are formed [5]. The transition from lamellar phase to cubic phase can be monitored by the optical property of the phase (loss of birefringence, see Figure 1). The depiction of the phases has been adapted from M. Caffrey [33]. The water content required for the formation of the individual phases can be targeted by vapor diffusion and addition of diluted screen solution as indicated (e.g. 1/1 means undiluted and 1/4 means the dilution of screen solution by a factor of 4).

As shown in the simplified phase diagram of monoolein (MO)/water at room temperature (Figure 2), these lipids – besides the cubic phase – form additional types of phases depending on the water:lipid ratio. In the presence of certain concentrations of PEG or Jeffamine [9]–[11] the formation of a dispensable sponge phase containing monoolein is favored. The requirement to use certain compounds to obtain this dispensable phase unfortunately limits the scope of this approach and compares unfavorably with the several thousands of different conditions that are used for the crystallization of soluble proteins. While membrane protein crystals can be obtained with different meso phases of the phase diagram, it is inherent to batch and the sponge phase methods to miss part of the space where high-quality crystals may form.

We therefore sought to develop a more flexibly applicable method for crystallizing membrane proteins *in meso*. A superior approach should (i) employ the equipment for high-throughput screening already in use for the crystallization of soluble proteins, which (ii) consequently requires to avoid the necessity to handle highly viscous lipids, it should (iii) allow to utilize the multitude of screening conditions commercially available to accommodate the hydrophilic parts of the membrane protein, (iv) embed the membrane part into a meso phase, (v) allow for controlled change of concentration by dehydration of the protein-lipid mixture to target specific meso phases, and (vi) should require only small quantities of protein but should allow to grow crystals in a timeframe of days to weeks.

We have developed protocols and materials to meet these demands by combining the *in meso* principle with vapor diffusion into a new method for controlled *in meso* phase crystallization (CIMP) of membrane proteins.

## Results

### Concept of CIMP crystallization

We investigated the well established crystallization of BR [12] and the also previously crystallized halorhodopsin [13] to establish suitable setup and starting parameters for a fast screening method for *in meso* crystallization of membrane proteins by vapor diffusion. The obtained procedures and parameters for the experiment were successfully tested by crystallizing the so far uncrystallized sensory rhodopsin II from *H. salinarum*. The color of the rhodopsins allows direct monitoring of the protein distribution in the meso phases. Detrimental effects of crystallization conditions on protein stability are detectable as loss of protein color due to release of chromophor. In order to allow the use of available liquid handling robotics for an automated reaction setup, we precoated the protein wells of crystallization microplates with monoolein lipid, dried the lipid and stored the plates in the absence of oxygen at −20°C after sealing under nitrogen.

At 22°C, the solid monoolein self-organizes with water into isotropic meso phase within 20–40 minutes. This self-organization into isotropic cubic phase can be observed optically as a loss of birefringence (Figure 1A–E). The isotropic cubic phase Pn3m consists of a bi-continuous bilayer that separates two channel systems of aqueous phase (Figure 2, meso phase structure, indicated by blue coloring). The membrane-like bilayer of monoolein is locally 2-dimensional like a cell membrane and therefore allows the incorporation of membrane proteins, but it extends continuously through space and therefore supports diffusion of the protein in three dimensions. Crystallization of embedded membrane protein is thought to occur upon formation of lamellar phase from cubic phase [12], [14]. In principle, this approach allows the accommodation of the hydrophobic regions of the membrane protein in an almost natural way into the bilayer. The hydrophilic regions of the protein exposed to the aqueous phase (water channels on both sides of the bilayer) are accommodated by suitable buffer compositions, which must be determined by buffer screening as it is the case for soluble proteins.

**Figure 3:**
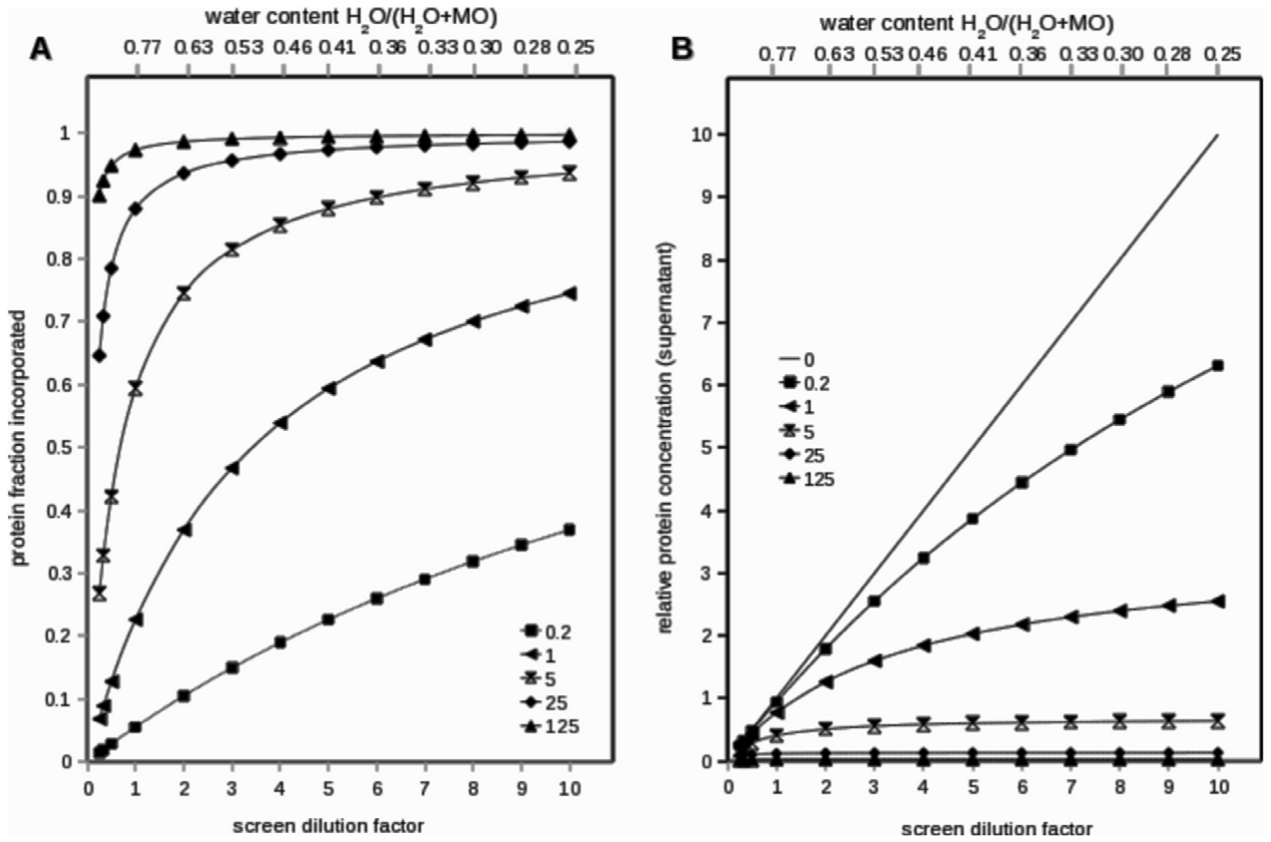
The effect of dilution for different partitioning constants K. **A**, Fraction of total protein incorporated into lipidic phase calculated for different partitioning constants as a function of water content (upper abscissa). **B**, Remaining concentration of protein in the aqueous phase (supernatant including the water content of the mesophase) relative to sample concentration calculated for different partitioning constants as a function of water content (upper abscissa). The lower abscissa gives the dilutions of the screening solution added to perform the standard experiment targeting the hydration level indicated on the upper abscissa. Dilution factor 1 refers to undiluted screening solution added, factor 2 refers to a 1∶2 diluted screening solution etc. Standard experiment: 132 µg monoolein plus 450 nl protein solution (sample) plus 450 nl undiluted or diluted screening solution to be equilibrated against undiluted screening solution in the well reservoir. Calculations are based on a partitioning model, where the protein is assumed to be monomeric in both phases with K = C_lip_/C_aq_, ,where C (molality) refers to protein concentration in the lipidic or aqueous phase. Courses of lipid-incorporated and protein remaining in the aqueous phases in A and B are depicted for K values between 0 and 125 as indicated in the insets.

In vapor diffusion experiments, a droplet of a volume of protein solution mixed with a volume of a solution containing a precipitating agent is equilibrated against a larger reservoir of the undiluted precipitating agent to achieve super saturation by transfer of water from the protein droplet to the reservoir through the vapor phase. The final volume of the protein droplet is determined to first approximation by the equality of precipitating agent concentrations in the condensed phases. Therefore, the final volume of the crystallization droplet is determined by the amount of precipitating agent added. During equilibration the reduction of the protein droplet's volume leads to an increase of protein concentration (s. curve K = 0 in Figure 3B).

In our approach, equilibration by vapor diffusion in the presence of monoolein not only allows varying the expected final protein concentration but also the total amount of water in equilibrium with monoolein. The latter determines the type of meso-phase, whereas the former determines, by the Nernst partition law, the distribution of protein between the aqueous and the forming meso-phase. Decreasing amount of available water forces the lipidic meso-phase phase to adopt a meso phase structure with less water (Figure 2). Decreasing amount of available water also forces the protein into the meso-phase. In the limiting case of the total disappearance of the aqueous phase, all protein must be incorporated into the lipidic phase.

**Figure 4:**
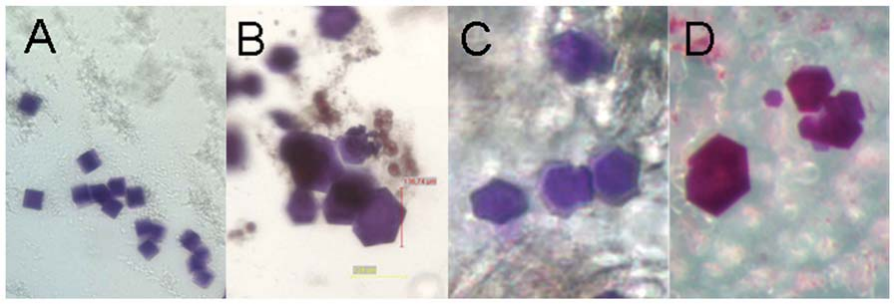
CIMP crystallization of BR (*H. salinarum*). **A**, Crystals from high excess water crystallization condition after 2 weeks (expected hydration level 75%). Ammonium sulfate was used as precipitant. Crystal size is approximately 11 µm. **B**, Crystals from excess water crystallization condition after 5 days (expected hydration level 60%). Na/K phosphate was used as precipitant. Crystal size is approximately 140 µm. **C**, Crystals from cubic phase crystallization conditions after 10 weeks (expected hydration level 43%). Na/K phosphate was used as precipitant. Crystal size is approximately 100 µm. **D**, Crystals from cubic/lamellar phase crystallization conditions after 14 weeks (expected hydration level 30%). Na/K phosphate was used as precipitant. Crystal size is approximately 200 µm.

If such an experiment is performed, transformation of meso phases and integration of the membrane protein into the lipidic phase are observed, and under suitable conditions this leads to the formation of protein crystals within the meso phase (Figure 4). Specific meso phases can be targeted by sets of such experiments with different final hydration levels, e.g., by employing undiluted (1∶1) and 1∶4 and 1∶7 dilutions of screening solution (Figure 2, Figure 3). Thereby, the explicit determination of the phase diagrams of monoolein, which depend on the used precipitant and detergent as well as other additives, can be avoided.

The distribution of protein between the aqueous and the lipidic phase under our standard conditions was measured and calculated, resulting in partitioning constants K, whereas a high K value indicates a high degree of protein incorporation into the lipidic phase. Figure 3A shows that for partitioning constants K>5 the expected incorporation into the lipidic phase exceeds the 90% threshold for water contents smaller than 40%. Furthermore, the protein concentration in the supernatant will not exceed the concentration of the protein in the protein sample used in the experiment if the removal of water from the experiment is slower than the speed of incorporation of the protein into the lipidic phase (Figure 3b). If one of these conditions is not met precipitation of the protein from the aqueous solution in the crystallization experiment would be possible. For partitioning constants K<0.2 the incorporation becomes less than 30% of total protein, which would severely impair the utility of CIMP crystallization (Figure 3b). For bacteriorhodopsin, apparent partitioning constants in the range of 13 to 130 were found by measuring the protein concentration in the aqueous supernatant.

In the case of slow integration into the lipidic phase or a small K value, the increase of the protein concentration during equilibration by vapor diffusion will at most reach the values calculated for ideally diluted conditions with K = 0 (Figure 3b). In general, it is expected that an increase in protein concentration increases the speed of mass transfer of protein into the meso phase and thereby limits the increase of the protein concentration in the aqueous phase during the removal of water by vapor diffusion.

**Figure 5:**
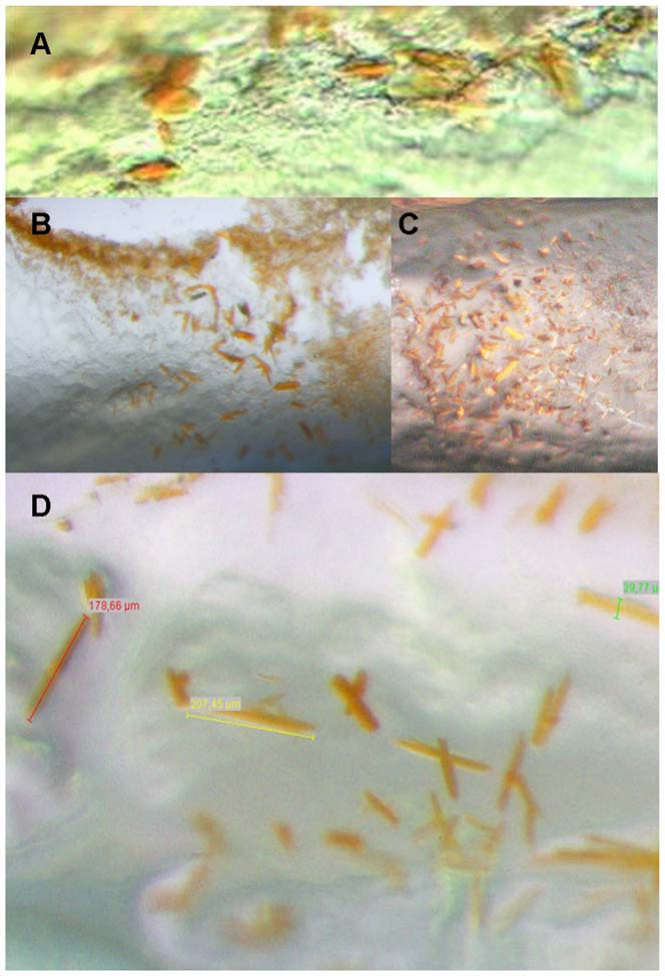
CIMP crystallization applied to Sensory Rhodopsin II (*H. salinarum*). SRII crystals from cubic phase using the precipitant ammonium sulfate ((NH_4_)_2_SO_4_) are shown. **A**, First hit after 12 weeks incubation time, expected final (initial) hydration level was 43% (87%). 4.0 M (NH_4_)_2_SO_4_ was used as precipitant. Crystal size is approximately 6 µm. **B**, First diffracting crystal obtained after 3 weeks incubation time, expected final (initial) hydration level was 43% (87%). 3.3 M (NH_4_)_2_SO_4_, 50 mM malonate was used as precipitant. Crystal size is approximately 140 µm. **C**, Optimized crystallization condition, expected final (initial) hydration level was 30% (87%). 3.3 M (NH_4_)_2_SO_4_, 600 mM malonate was used as precipitant. **D**, Optimized crystallization condition, expected final (initial) hydration level was 30% (87%). 3.3 M (NH_4_)_2_SO_4_, 50 mM malonate was used as precipitant. Crystal size is approximately 180 µm.

When a high protein concentration is used or when the mass transfer from the aqueous supernatant into the surface of the lipidic phase is much faster than the diffusion of the protein within the cubic phase, crystallization may occur before the protein is homogenously distributed within the meso phase (Figures 5B, S1). In principle, this allows screening different protein concentrations within one experiment.

### Parameters and phases in CIMP crystallization

The typical CIMP crystallization experiment has three distinct phases, i) the swelling phase when solid monoolein takes up aqueous solution (Figure 1) to form cubic phase, ii) the equilibration phase during which the meso phase is dehydrated, and iii) the incubation phase when the hydration level of the meso phase remains constant and the only remaining process is protein diffusion and possibly crystallization (e.g., Figure 5D).

To start the *swelling phase* (monoolein hydration), we first tested the solid monoolein in the crystallization plate with various volumes of protein solution. The solid monoolein with 86% water transforms completely into isotropic phase within 40 minutes (Figure 1A–E). If a solution of detergent-solubilized membrane protein is used, the phase transformation time is considerably prolonged and variable, even in the presence of excess volume of aqueous solution. The competing reaction, the uptake of monoolein by the aqueous solution, is typically of minor importance because of the low solubility of monoolein in water. For the precoated plates with 132 µg (29 µg) of monoolein, we found a volume of 900 nl (200 nl) (Figure S2) of aqueous, i.e. protein plus screening solution sufficient to completely wet and hydrate the surface of the solid monoolein at 22°C. Although the actual minimal volume varied depending on the composition of the protein solution, we found 900 nl (200 nl) to be sufficient in all cases to obtain isotropic (cubic) mesophase.

The immediate addition of screening solution to the crystallization well after dispensing highly concentrated protein solution onto the dry monoolein has been evaluated with BR. This modified procedure would allow one to avoid plate handling steps and to skip the swelling phase and save 3 hours of incubation time. Colored crystals of BR were indeed obtained but they were tiny (∼5 µm) and of minor quality (data not shown). In further experiments, we observed that highly concentrated protein solutions can precipitate under these conditions, and induce crystallization of membrane protein in aqueous solution (Figure 4A). Therefore, we started experimental series generally with a swelling time of three hours prior to adding the screening solution to the protein well.

In the *equilibration phase* (monoolein dehydration), the mixture of protein and screening solution in the presence of monoolein is equilibrated against screening solution in the reservoir. Within days to weeks, equilibration of the vapor diffusion experiment occurs. The *incubation phase* (protein diffusion) starts once the equilibrium condition of the vapor diffusion experiment has been reached. Diffusion-controlled crystallization of the protein in the lipidic matrix may occur within weeks to months (Figure 4C, 4D, 5D). In any case, we did not observe a crystallization time that exceeded 14 weeks. The workflow of the developed *in meso* crystallization method is very similar to the procedure of a regular vapor diffusion setup (Figure S2).

### CIMP crystallization experiments with Bacteriorhodopsin and Halorhodopsin

BR was used as the model protein to establish and optimize the CIMP crystallization method. The optimization of the crystallization conditions was based on the information that BR is crystallizable using phosphate as dehydrating agent. In a grid screen (pH 4.5 to 8.5, 0.5 M to 4.0 M phosphate) [3] with 8 plates we varied the protein concentrations, the initial droplet volume and, by dilution, the concentration of screening solution in the initial droplet. Twinned crystals that diffract to a resolution of 1.3 Å were obtained (Figures S3, 4B). Data obtained at the ESRF in Grenoble (ID29) were useful to a resolution of 1.45 Å (Rmerge = 0.065) with a Mean (I/sigI) of 3.3 for the resolution shell 1.54–1.45 Å. As expected, no significant differences to the known high-resolution structure of bacteriorhodopsin from also twinned crystals (1C3W) [15] were observed.

Further crystallization experiments with other screens we designed for the LCP method (salt versus pH, the CubicPhase I grid screen, and PEG versus pH, the CubicPhase II grid screen) and further salts failed for BR with the notable exception of ammonium sulfate. With this salt, we obtained non-diffracting crystals (Figure 4A) of cubic appearance (targeted phase Pn3m with excess water, Figure 2). Crystals of this appearance had been obtained earlier in the absence of mesophase also with ammonium sulfate from aqueous solution of the protein. This condition is known not to produce well diffracting crystals [16]. It is noteworthy that these crystals can be obtained with neither ammonium phosphate nor sodium/potassium phosphate. We conclude that interactions between the salt and BR are required to induce crystallization, an effect that has previously been proposed in complete generality [2].

Although crystallization of BR has been streamlined to yield high-quality crystals (Figure 4B, Figure S3), it can even be forced to occur during the equilibration phase upon overnight incubation of the experiment if conditions have been optimized accordingly (Video S1). This allows continuous recording of the progress of the crystallization experiment even though the obtained crystal size appears to be adversely effected by the required continuous illumination. This allowed for the first time to visualize and document the complete course of membrane protein crystallization in meso phase. The time-lapse movie revealed that (i) the passive incorporation of BR into the mesophase generates a lateral protein concentration gradient, (ii) transformation of cubic phase occurs first where the protein and the detergent concentration is highest, and that (iii) crystallization may occur at a protein concentration that is visually undetectable by the red color of BR, which indicates that here the amount of protein per area is much lower than in the red colored region. These observations imply that an optimal condition (ratio protein:monoolein:detergent) exists, which may be more difficult to find in batch-LCP crystallization approaches.

The same condition that allowed over-night LCP crystallization of BR was applied to a different format: hanging drop vapor diffusion crystallization using a sealing foil and a standard micro titer plate (Figure S4). The same workflow as for sitting-drop experiments described so far (Figure S2) was followed. Crystals of BR of up to 50 µm in size were generated after 16 hours incubation. Similar to the observation made in the time-lapse movie (Video S1), crystals mainly grew in the periphery of MO/protein droplets. The hanging drop variant seems to work equally well as the sitting-drop method and may be used to further simplify the procedure of LCP crystallization by CIMP crystallization.

A setup similar to BR was started in the sitting-drop mode for halorhodopsin (HR) based on published crystallization conditions [13]. Small crystals diffracting to a resolution of 8 Å were obtained within the first three plates that were set up. Variation of the ß-octylglycoside concentration by adding 1% (w/v) detergent to the screening solution improved the crystal size to 0.27 mm (Figure S1). Optimization of HR crystallization was not investigated further.

### CIMP experiment with *H. salinarum* Sensory Rhodopsin II

Instead, we investigated *H. salinarum* Sensory Rhodopsin II (SRII), which had not been crystallized before. We failed to obtain crystals using the crystallization conditions of the more stable homologous protein from *N. pharaonis* [17]–[19]. Therefore, we tested the effect of different compounds on solubility and stability of the protein in detergent solution with a commercial pre-screen assay kit. The protein was stable in four unbuffered respectively neutral solutions (4.3 M NaCl, which is close to the purification condition, 1.1 and 3.2 M ammonium sulfate, and 3 M Na/K phosphate, pH 7.0), whereas in all other cases the protein precipitated immediately or latest after one day. Consequently, we set up trials in Na/KPO_4_ and ammonium sulfate (from 1.6 up to 4.0 M) for crystallization in the CubicPhase micro plate.

After three months, crystals with a size of 5–10 µm appeared in the highest concentration of ammonium sulfate (Figure 5A). In the next setups, we combined the ammonium sulfate screen with different additives by mixing it with commercial anion-, cation-, PEG-based or the Optisalts screening suites by diluting the precipitant with additive to obtain a final additive solution content of 10% (v/v). After a two months incubation time, we could identify sodium malonate from the Optisalts screen as the one additive that led to improved crystal size.

Further optimization (fine screening) of protein concentration, mixing ratio (dilution factor) and reservoir concentration led to a reduction of the crystallization time to 3 days.

Best diffracting crystals (40×40×250 µm) have been obtained after 10 to 17 days by equilibrating 450 nl protein solution (highly pure protein preparation of 31 mg/ml with *H. salinarum* polar lipids in a ratio 10∶1 mol/mol protein) plus 450 nl of unbuffered 1.7 M (NH_4_)_2_SO_4_, 190 mM sodium malonate (1∶2 dilution of the reservoir solution) against 3.4 M (NH_4_)_2_SO_4_, 380 mM sodium malonate in the reservoir. Data obtained at the ESRF, Grenoble (ID 14) were useful to 3.5 Å (Rmerge = 0.129) with a Mean(I/sigI) of 3.4 for the resolution shell 3.69–3.50 Å. The structure SRII of *H. salinarum* has been solved and will be published elsewhere.

## Discussion

We have developed a novel LCP technology for controlled *in meso* crystallization (CIMP) of membrane proteins which should prove useful to overcome current limitations associated with structural investigation of this type of proteins. CIMP has been applied successfully to crystallize several integral membrane proteins, and we report the crystallization of one of those: SRII from *H. salinarum*, a previously uncrystallized light receptor. Major problems in membrane protein crystallization employing traditional methods are caused by the low solubility of membrane proteins that must be overcome by a detergent that increases the amount of dissolved protein substantially without destabilizing it. We resolve the solubility problem by the application of the vapor diffusion method, which allows increasing the protein concentration by removing excess water through the vapor phase. This advantage is also given for the dispensable sponge phase crystallization approach [10], even though its applicability is limited by the number of compatible screening solutions. However, it is missing in the pioneering approach of Landau and Rosenbusch [3]. With CIMP crystallization, the ratio of membrane protein:meso phase is limited only by the amount of protein (and detergent) that the mesophase can incorporate. CIMP crystallization not only enables a very fast and complete screening of the parameter space for crystallization in combination with robotic systems, but also allows to reach parts of the parameter space that are principally inaccessible by batch methods. CIMP crystallization varies concentration of protein in the lipidic phase and the precipitating agent in the aqueous phase within the equilibration period of the experiment as typical for vapor diffusion experiments, which is not the case for LCP crystallization from pre-prepared protein-containing mesophase paste where protein and additive concentrations hardly change at all. Furthermore, the generation of a spatial gradient of protein within one experiment is only possible with a method such as CIMP that uses passive mixing. This is achieved by pre-incubation of the solid monoolein with the protein solution for about 3 hours (Figure S2, Video S1).

It should be noted, however, that all components of the protein solution will be concentrated during the vapor diffusion experiment [1], especially if a dilution scheme as described in Figure 3 is applied. For example, in the case of halorhodopsin, the presence of a second detergent is a critical parameter for crystallization success. In such a case, however, where a certain additive needs to be present, an adaption of this condition can be easily achieved by including the additive in the diluted screening solution that is added to the crystallization droplet.

The complex composition of aqueous solutions used in the crystallization experiments implies that the simple phase diagram of the monoolein/water system (Figure 1) insufficiently describes the more complex system used here. In any real experiment, the phase diagram of the aqueous monoolein system will be unknown because, in addition to water and monoolein, also protein, buffer, salt, detergent [20], and lipids [21] will be present and influence the phase stability and transformation. Known phase diagrams can only define starting parameters for the crystallization experiments. Nevertheless, we found the monoolein-water phase diagram sufficient to target the different mesophases by using different dilutions of screening solution (Figure 3).

The recommended protocol (Figure S2, see also Material and Methods) gives the extreme or best values of the experimental parameters, e.g., with respect to the volume of the initial aqueous phase of 450 nl+450 nl: the total volume of 900 nl per 132 µg monoolein is the minimum required to obtain reasonable lipid hydration, compactness and surface smoothness of the resulting mesophase, which allows to detect even colorless crystals (Figure S5A). The volume of protein solution can be increased at will to increase the protein:monoolein ratio. The added volume of screening solution, however, should be kept constant as it is an optimal parameter that allows, for different dilutions of screening solution, to target the different meso phases.

We found that our approach as outlined can be easily scaled down to 29 µg of monoolein per experiment which corresponds for the optimize crystallization conditions to a consumption of 1.6 µg of protein (100 nl; 16 µg/µl) per BR crystallization experiment. BR crystals obtained under these conditions were about 30 µm in size. Crystals of this dimension can be analyzed on all micro-focus synchrotron sources. Experiments with 132 µg MO (7.2 µg protein, 450 nl solution) led to crystals of 200 µm in length under the same conditions. However, our very early experiments using 500 µg MO did not yield crystals larger than 200 µm. Therefore, the scalability of the crystal size that can be obtained from LCP experiments seems to be limited.

In the described CIMP crystallization approach, the kinetics of all processes before reaching equilibration is ultimately driven by the equilibration through the vapor phase. In some cases, it may be desirable to slow down equilibration to allow more time for the protein to migrate within the mesophase. Although none of our test proteins required this for crystallization, the equilibration of the vapor diffusion experiment can be slowed down easily by adding a layer (of, e.g. mineral oil) on top of the reservoir solution [22], [23].

Upon recording BR crystallization experiments through a microscope we observed that the colored protein becomes inhomogenously distributed within the mesophase (Video S1). Protein crystallization was seen first in a region of low protein concentration (Video S1, Figure S4), which seems to be ideal for initiation of this event. We believe that crystal growth is supported by continued supply of protein from regions of high protein concentration. We regard it as one of the big advantages of the CIMP method to have an inhomogeneous protein distribution within the mesophase as this allows, in principle, to screen at any point in time different protein concentrations within the same experiment. The formation of a concentration gradient for the membrane protein appears to be more pronounced the higher the initial protein concentration was. It is noteworthy that such gradients can also be observed in the classical mesophase experiment with solid salt.

A major concern with regard to the crystallization of temperature sensitive proteins is the required incubation temperature of at least 18°C for monoolein phases [7]. But even for a less stable mammalian GPCR, crystallization in mesophase was reported [24], [25]. Clearly, membrane proteins embedded in meso phases exhibit an increased half-life compared to the detergent-solubilized state [26]. Nevertheless, a reduction of the required incubation temperature is desirable and may be attempted by doping the mesophase with lipids like cholesterol or by the exchange of the major component monoolein [26]–[28].

The experiments shown, e.g., in Figure 5D and Video S1 have been repeated more than 20 times and always gave the same results; moreover, CIMP crystallization has been performed with different protein batches of BR and SRII and in different laboratories with varying equipment (data not shown). Therefore, we regard the reproducibility and robustness of CIMP crystallization as very high.

Currently the protein consumption per experiment with 29 µg of monoolein requires 100 nl of protein solution whereas the paste method requires 20 nl per 30 µg [7]. As, however, diluted protein solution can be used for CIMP crystallization, the current consumption of these methods is about equal when equal amounts of protein per monoolein are targeted. In fact, the protein consumption of CIMP crystallization can be considerably lower because of the gradual increase of local protein concentration by protein and vapor diffusion. We obtained crystals of bacteriorhodopsin with a size of 3–5 µm using concentrations as low as 1.6 mg/ml (0.7 µg per experiment with 132 µg monoolein) within 48 hours. Interestingly, the crystallization time can be also considerably lower than for the paste method. We obtain crystals deeply embedded into meso phase after 10 to 14 weeks (Figure 4 C,D) , whereas crystals close to or at the water/lipid phase boundary form within hours (Video S1) or days (Figure 4B). We suggest that the diffusion of protein molecules within the meso phase is much slower than the incorporation of the protein into the surface of the meso phase when using CIMP crystallization employing an adequate setup. This would lead to the nucleation at lower overall concentration of protein than observed for the paste method. Crystal growth could be fed by protein from the aqueous solution. In any case, such a setup should be realizable when employing a diffusion barrier as described.

CIMP crystallization using precoated plates requires no steps that cannot be easily implemented using standard robotics, which is not the case for the LCP approaches reported so far. We believe that the CIMP method can contribute to accelerate the progress in crystallization and structure determination of membrane proteins.

## Materials and Methods

### Reagents, solutions, and crystallization microplates

96-well crystallization plates precoated with monoolein (CubicPhase µplates) with 500 µg, 132 µg or 29 µg of monoolein per experiment and screening suites (CubicPhase I, CubicPhase II, Optisalt, Easy Xtal Pre-Screen Assay) as well as Ni-NTA Agarose were obtained from Qiagen. Detergents were from Glycon.

**Crystallization plates coated with 500** µg and 150 µg monoolein were initially prepared by dispensing molten monoolein at 42°C. This dispensing procedure was found to be unreliable for 150 µg coatings. Precoated plates from industrial production with 132 µg or 29 µg are produced with CV values of less than 5%.

### Protein expression and purification

Bacteriorhodopsin (BR) was prepared as described previously [29].

SRII was expressed in *E. coli* and purified in 4.0 M NaCl, 50 mM MES, pH 6, 0.05% DDM (n-dodecyl-ß-maltoside) as described previously [30].

His-tagged halorhodopsin (HR) was expressed in *H. salinarum*. The HR overexpressing L33 strain [31] was shaken under illumination for 2–4 days at 120 rpm in a medium containing 1% (w/v) peptone, 4.3 M NaCl, 80 mM MgSO_4_×7H_2_O, 27 mM KCl, 10 mM tri-NaCitratx2H_2_O, pH7.0. At OD_600 nm_ 1–1.5, shaking speed was reduced to 80 rpm for 2–4 days. Cells were harvested and resuspended in 100 mM sodium phosphate buffer, pH 7.2, supplemented with DNase I, and passed three times through an EmulsiFlex-C3 homogenizer (Avestin). The insoluble fraction which contains halorhodopsin was isolated by centrifugation (130,000× g, 1 h, 4°C) and solubilized overnight at 4°C in 4.0 M NaCl, 50 mM MES, pH 6.0, 2% (*w*/*v*) DDM. The solubilized membrane protein fraction was isolated by centrifugation (130,000× g, 1 h, 4°C) and the target protein purified by Ni-NTA Agarose chromatography in 4.0 M NaCl, 50 mM MES, pH 6.0, 0.05% (*w*/*v*) DDM. Detergent was exchanged by washing extensively with 4.0 M NaCl, 50 mM MES, pH 6.0, 0.1% (w/v) ß-octylglycoside. The protein was eluted by lowering the pH to 4.5. The protein was stored at pH 5.5–6.

Purified membrane proteins were concentrated by ultra-filtration (Molecular weight cut-off 10 kDa) and filtered (0.22 µm, Millipore) as described [32].

### Determination of apparent partitioning K

Monoolein precoated plates were incubated with various volumes of protein solution of known concentration. After 24 hours of incubation at 22°C the supernatant was separated by aspiration and centrifuged at 20000 g for 10 minutes. The protein concentration in the obtained supernatant was photometrically determined. The K value is calculated according to the information given in the legend of Figure 3.

### Optimized crystallization protocol (standard conditions)

Microplates precoated with 132 µg (29 µg) monoolein and stored at −20°C were thawed at 22°C for 10 minutes. 450 nl (100 nl) of protein solution per experiment is dispensed on top of the MO coating to start MO swelling and the formation of cubic phase. The plate is sealed and incubated at 22°C. After 3 hours, 450 nl (100 nl) of screening solution diluted, e.g., 1∶4 (Figure S2) are added to the experimental well and 75 µl of undiluted screening solution is transferred to the reservoir wells. The resealed plate is incubated at 22°C and monitored for progress (phase transformation and crystal formation) at least once per week.

Crystallization experiments using plates precoated with 132 µg MO were prepared manually or with Evo-100 Robot (Tecan), plates precoated with 29 µg and foils precoated with 100 µg monoolein were processed with a Mosquito robot (TTP-Labtech).

In foil-based hanging-drop crystallization, dry MO spots (100 µg each) were hydrated with 300 nl BR solution, incubated for 3 hours at 22°C on top of an empty standard 96-well micro plate (droplets hanging under the foil); then, the foil was removed, the precipitant solutions added to the micro plate wells, the foil placed back on top of the plate and incubated at 22°C for crystallization.

### Monitoring of protein crystallization

The phase transformation of monoolein was monitored under a microscope (SZX10, Olympus) for change in birefringence of the mesophase. For colorless crystals, the discrimination between speckles of lamellar phase and protein crystals becomes increasingly difficult with decreasing size of crystals. Therefore, we choose an UV-transparent material for the crystallization plates that allow monitoring crystal formation and detection of salt crystals using a fluorescence microscope (Figure S5).

## Supporting Information

Figure S1 **Crystallization of halorhodopsin (HR) from** ***H. salinarum*** **by CIMP.** Crystals 240 µm in size were obtained from cubic phase condition after 3 days. The final MO hydration level is estimated to be approximately 43%. The HR protein was crystallized using a precipitant mixture of KCl and ß-Octylglycoside.
(TIFF)

Figure S2 **Workflow for** ***in meso*** **crystallization by vapor diffusion.** The graphic flow scheme describes the standard experiment. It covers the swelling phase (self -organization of the mesophase) and the early equilibration phase starting with the addition of screening solution to the reservoir (R, undiluted) and to the protein droplet.
(TIFF)

Figure S3 **Bacteriorhodopsin diffraction.** Data were collected at ID29 at the ESRF (Grenoble) from the crystal shown in Figure 4B. The right panel shows a zoom into the region marked by the grey insert in the left panel.
(TIFF)

Figure S4 **Hanging-drop crystallization of BR by CIMP.** 100 µg of monoolein was spotted onto the lower side of hydrophobic and glue-free slots of a crystallization foil and subjected to hydration by 300 nl of BR solution (16 µg/µl). For swelling, the foil was placed over an empty standard 96-well microtiter plate for 3 hours at 22°C with the MO/protein spot hanging below the foil into the plate well. After incubation, the foil was removed from the microtiter plate, 300 nl of screening solution (2.8 M Na/K phosphate, pH 5.9) diluted 1∶4 was added to the MO/protein droplet and 50 µl of undiluted screening solution was filled into the corresponding well of the microtiter plate. The foil was placed back over the plate and incubated for crystallization over night. **A**, Close-up to well number A1 of the microtiter plate sealed with the crystallization foil. The purple color represents BR protein and protein crystals. **B**, Close-up to the MO/protein spot shown in **A**. BR crystals are visible in the periphery of the spot in the area of low protein concentration. **C**, Close-up to the upper third part of the spot shown in **B**. BR crystals of up to approximately 50 µm are generated after overnight crystallization. The bar indicates a size of 50 µm.
(TIFF)

Figure S5 **Crystal Detection.** Sensory rhodopsin crystals lost the chromophore under certain conditions and after long incubation. Colorless crystals with a size of 10–30 µm can be clearly observed by optical microscopy (**A**) and epifluorescence (**B**).
(TIFF)

Video S1 **Time course of CIMP crystallization of bacteriorhodpsin (BR).** For t = 14 hours, the experimental progress was recorded (Olympus SZX10, Olympus camera DP20) under constant cold illumination by glass fiber optics (Schott KL1500, Osram 150 W, Settings: half light, power level 1) to avoid uncontrolled changes in temperature. A brief script of what the movie shows is provided: at t = 0, swelling of 150 µg monoolein was initiated by dispensing of 450 nl BR protein solution (16 mg/ml). At t = 3 hours, 450 nl of 1∶4 dilution of reservoir solution were added to the protein well and equilibrated against undiluted reservoir solution (2.8 M Na/K phosphate, pH 5.8). Swelling continues and passes into equilibration phase. A BR protein concentration gradient from left to right can be clearly observed by the intensity of the purple color (left: high BR concentration; right: low BR concentration). At t = 6:30 hours, the microscope is refocused. At t = 7:45 hours, a local collapse of the cubic phase is observed in the centre of the well. After 10 hours, first crystals become visible on the right, the area of lowest protein concentration. At t = 10:30 hours, the movie zooms into the crystallization area. Crystals keep on growing until the end of the movie at t = 14 hours. The largest BR crystal visible in the time-lapse movie is approximately 30 µm.
(AVI)
