# Supplementary figures and images for: Controlled In Meso Phase Crystallization – A Method for the Structural Investigation of Membrane Proteins
Source: PLoS One. 2012 Apr 19;7(4):e35458. doi: 10.1371/journal.pone.0035458 (PMC3334905; doi:10.1371/journal.pone.0035458)

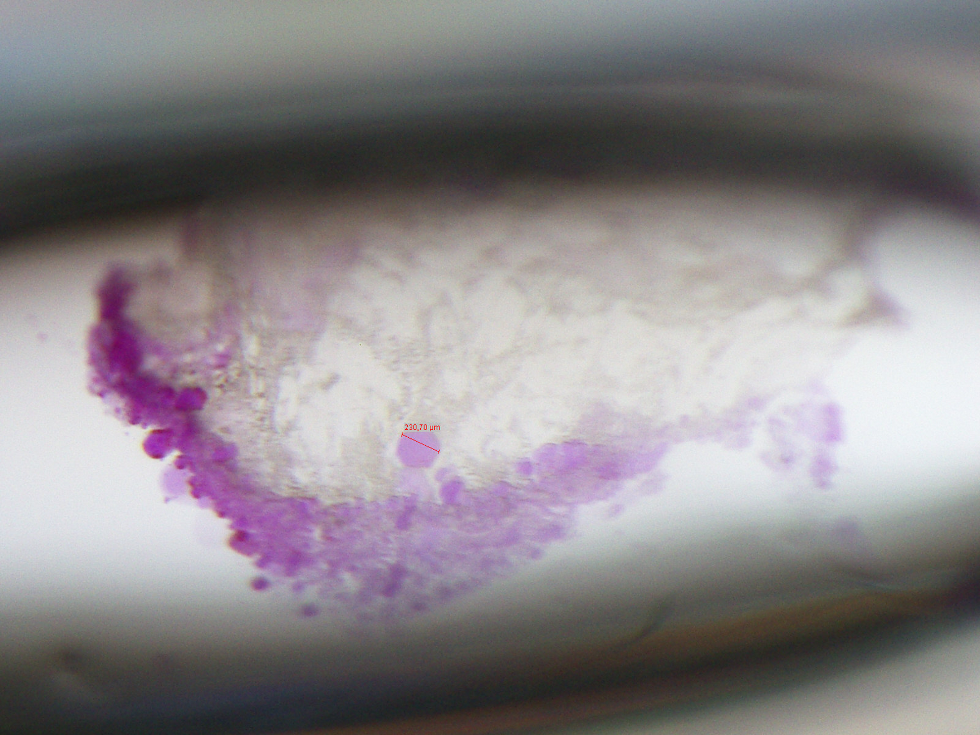

Supplement: Figure S1 — Crystallization of halorhodopsin (HR) from H. salinarum by CIMP. Crystals 240 µm in size were obtained from cubic phase condition after 3 days. The final MO hydration level is estimated to be approximately 43%. The HR protein was crystallized using a precipitant mixture of KCl and ß-Octylglycoside. (TIFF) [file pone.0035458.s001.tif]

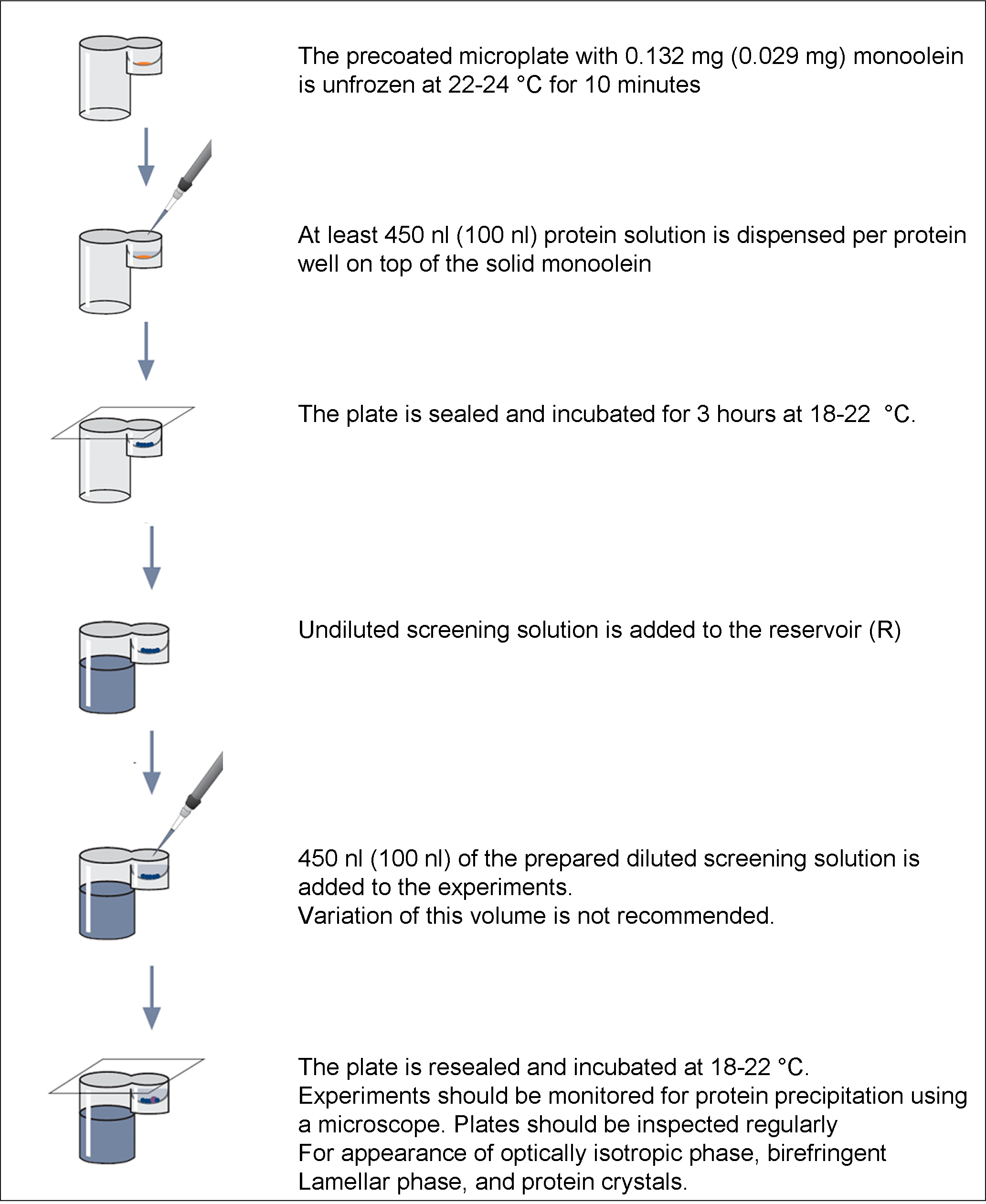

Supplement: Figure S2 — Workflow for in meso crystallization by vapor diffusion. The graphic flow scheme describes the standard experiment. It covers the swelling phase (self -organization of the mesophase) and the early equilibration phase starting with the addition of screening solution to the reservoir (R, undiluted) and to the protein droplet. (TIFF) [file pone.0035458.s002.tif]

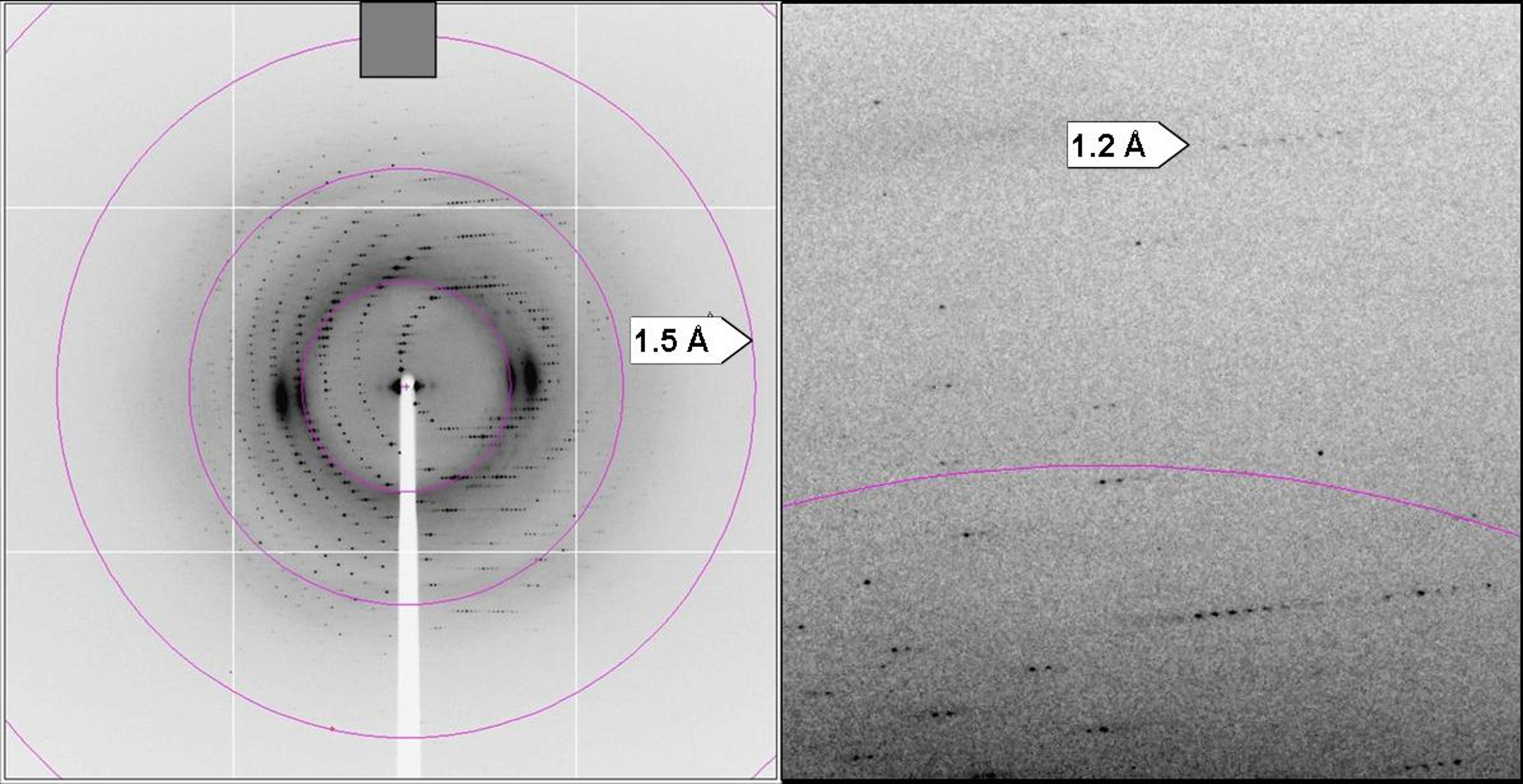

Supplement: Figure S3 — Bacteriorhodopsin diffraction. Data were collected at ID29 at the ESRF (Grenoble) from the crystal shown in Figure 4B. The right panel shows a zoom into the region marked by the grey insert in the left panel. (TIFF) [file pone.0035458.s003.tif]

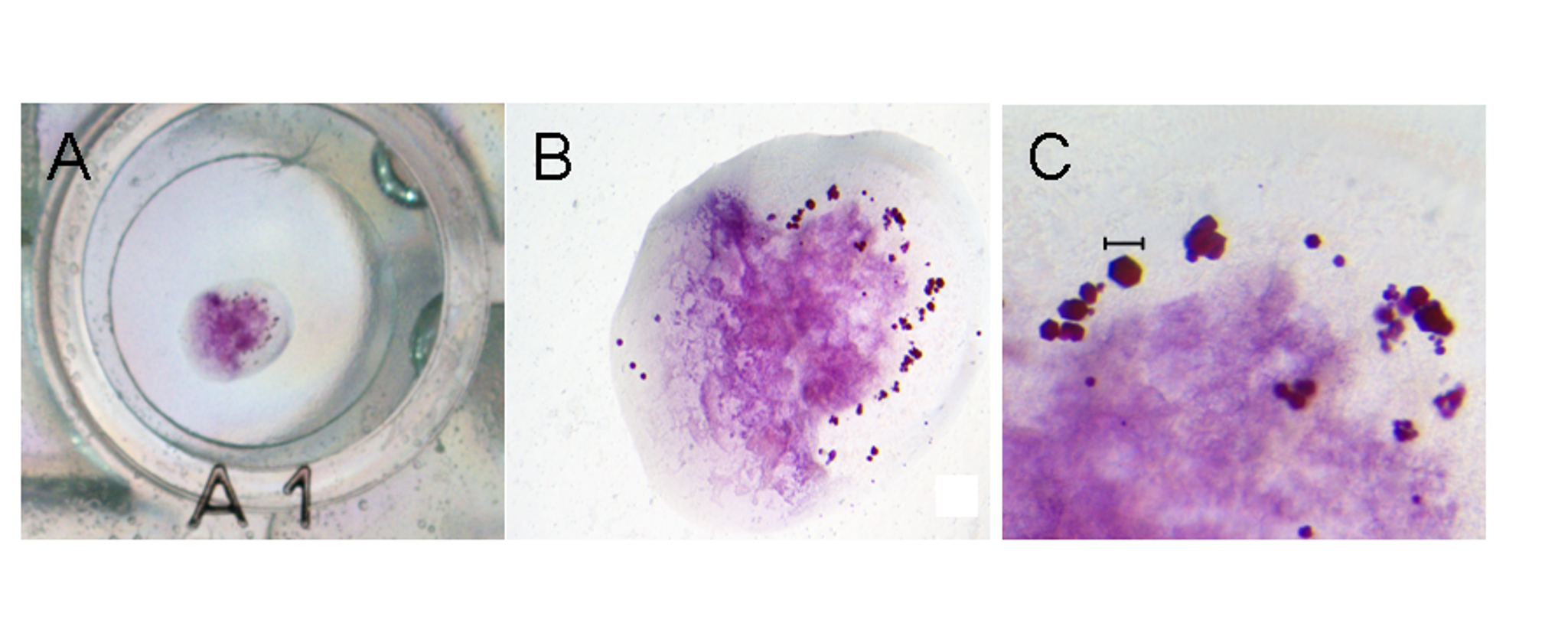

Supplement: Figure S4 — Hanging-drop crystallization of BR by CIMP. 100 µg of monoolein was spotted onto the lower side of hydrophobic and glue-free slots of a crystallization foil and subjected to hydration by 300 nl of BR solution (16 µg/µl). For swelling, the foil was placed over an empty standard 96-well microtiter plate for 3 hours at 22°C with the MO/protein spot hanging below the foil into the plate well. After incubation, the foil was removed from the microtiter plate, 300 nl of screening solution (2.8 M Na/K phosphate, pH 5.9) diluted 1∶4 was added to the MO/protein droplet and 50 µl of undiluted screening solution was filled into the corresponding well of the microtiter plate. The foil was placed back over the plate and incubated for crystallization over night. A, Close-up to well number A1 of the microtiter plate sealed with the crystallization foil. The purple color represents BR protein and protein crystals. B, Close-up to the MO/protein spot shown in A. BR crystals are visible in the periphery of the spot in the area of low protein concentration. C, Close-up to the upper third part of the spot shown in B. BR crystals of up to approximately 50 µm are generated after overnight crystallization. The bar indicates a size of 50 µm. (TIFF) [file pone.0035458.s004.tif]

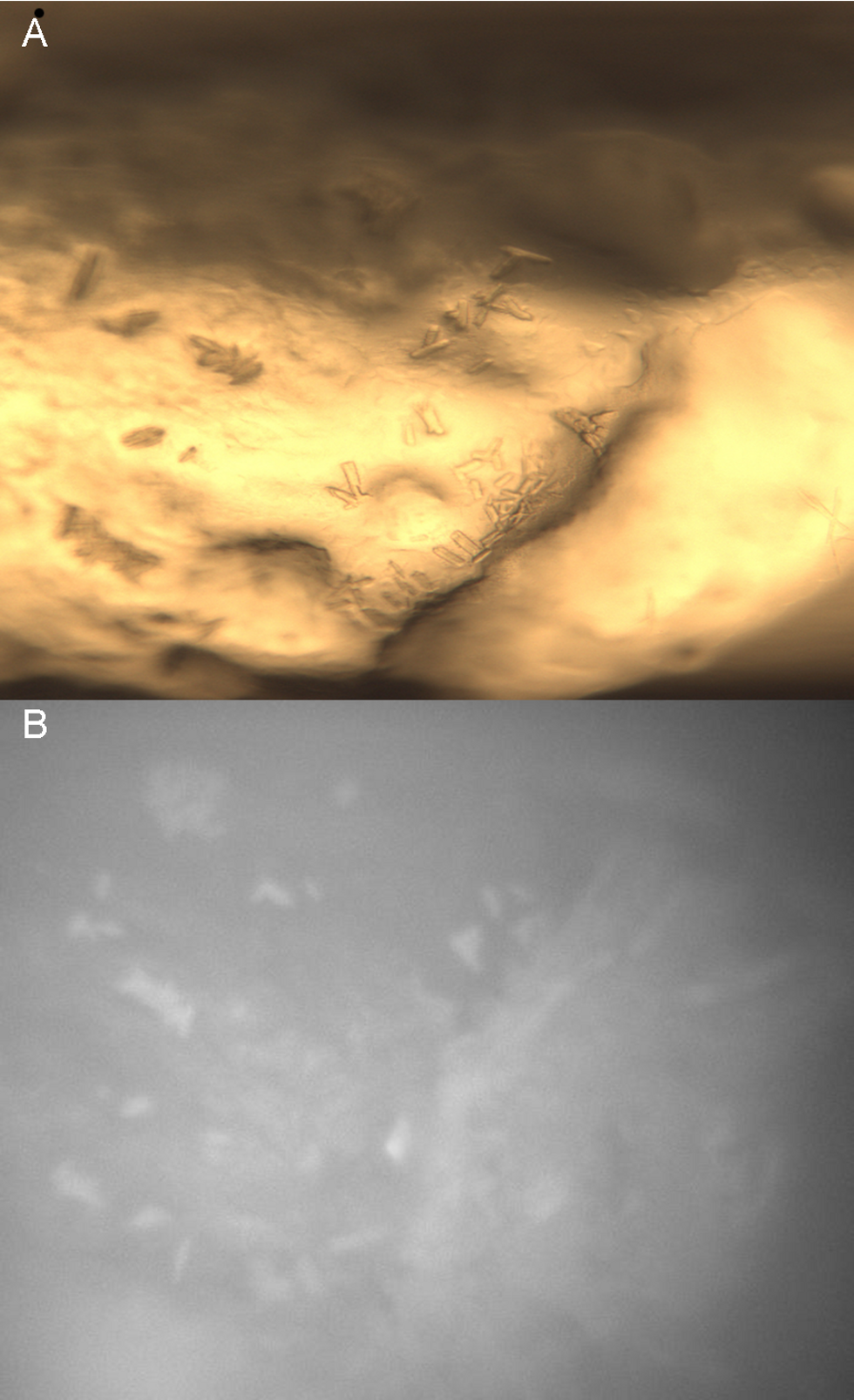

Supplement: Figure S5 — Crystal Detection. Sensory rhodopsin crystals lost the chromophore under certain conditions and after long incubation. Colorless crystals with a size of 10–30 µm can be clearly observed by optical microscopy (A) and epifluorescence (B). (TIFF) [file pone.0035458.s005.tif]
